# Thermal and mechanical characterization of high performance polymer fabrics for applications in wearable devices

**DOI:** 10.1038/s41598-021-87957-7

**Published:** 2021-04-22

**Authors:** Aaditya A. Candadai, Emily J. Nadler, Jack S. Burke, Justin A. Weibel, Amy M. Marconnet

**Affiliations:** 1 Birck Nanotechnology Center and School of Mechanical Engineering, Purdue University, West Lafayette, IN 47907 USA; 2 School of Mechanical Engineering, Purdue University, West Lafayette, IN 47907 USA

**Keywords:** Mechanical engineering, Polymers, Composites

## Abstract

With advances in flexible and wearable device technology, thermal regulation will become increasingly important. Fabrics and substrates used for such applications will be required to effectively spread any heat generated in the devices to ensure user comfort and safety, while also preventing overheating of the electronic components. Commercial fabrics consisting of ultra-high molecular weight polyethylene (UHMW-PE) fibers are currently used in personal body armor and sports gear owing to their high strength, durability, and abrasion resistance. In addition to superior mechanical properties, UHMW-PE fibers exhibit very high axial thermal conductivity due to a high degree of polymer chain orientation. However, these materials have not been widely explored for thermal management applications in flexible and wearable devices. Assessment of their suitability for such applications requires characterization of the thermal and mechanical properties of UHMW-PE in the fabric form that will ultimately be used to construct heat spreading materials. Here, we use advanced techniques to characterize the thermal and mechanical properties of UHMW-PE fabrics, as well as other conventional flexible materials and fabrics. An infrared microscopy-based approach measures the effective in-plane thermal conductivity, while an ASTM-based bend testing method quantifies the bending stiffness. We also characterize the effective thermal behavior of fabrics when subjected to creasing and thermal annealing to assess their reliability for relevant practical engineering applications. Fabrics consisting of UHMW-PE fibers have significantly higher thermal conductivities than the benchmark conventional materials while possessing good mechanical flexibility, thereby showcasing great potential as substrates for flexible and wearable heat spreading application.

## Introduction

The evolution of electronic devices toward thin, portable, and flexible systems has created new opportunities for device integration^1,2^. As a result, wearable technology has emerged as a means of providing a multifunctional infrastructure to facilitate various consumer needs^3,4^. In particular, interconnected devices such as smart textiles, wearable health monitoring systems, and wearable displays are becoming increasingly popular, and have the potential to revolutionize industrial sectors such as health care, personal computing, and sports^5–7^. Such applications pose new materials development and design challenges in order to realize electronic functionality and reliability in soft, flexible, and lightweight devices^8,9^. While there has been much work in the development of electrically conductive polymers and textiles^9,10^, there has been limited focus on the thermal management considerations associated with such applications.

Thermal management of wearable devices is a challenging problem driven by both ergonomic and electronics-reliability considerations. Electronic components need to be kept to within certain operating temperature limits dictated by their reliability, but the proximity of the wearable device to the human skin simultaneously requires maintaining the surface of the device at a comfortable temperature to touch^2,11,12^. When conventional (electrically insulating) polymers are used as substrates for electronic device attachment^13,14^, dissipation of the heat generated in these devices becomes a bottleneck for performance and reliability due to the low thermal conductivity of these polymers. Furthermore, consumer expectations pose size and weight constraints often desiring small form factors^15,16^ due to which incorporation of bulky heat sinks or cooling systems is not possible. Further, integration of the device into a wearable electronics package while ensuring user comfort requires mechanical flexibility. Thus, both the mechanical compliance and the intrinsic heat dissipation properties must be considered for substrate materials to be used in wearable electronic devices.

Many studies have focused on developing and characterizing the properties of thermoregulation textiles based on moisture wicking^17,18^, liquid cooling^19–21^, and radiation cooling mechanisms^22–24^. While moisture management and radiation cooling mechanisms are available for personal thermal management, they are less relevant for heat dissipation from wearable electronic devices. Active cooling strategies such as liquid-cooled garments can be bulky, expensive, and power consuming^24^. To overcome some of these limitations, recent work has focused on developing *thermally conductive* polymer-based textiles as substrates for thermal management^25–29^. For example, Gao et al.^25^ demonstrated 3D-printed fabrics constructed from aligned boron-nitride (BN)/poly-vinyl alcohol composite fibers for personal cooling applications with enhanced heat spreading properties and ~ 2 times higher thermal conductivity compared to conventional textiles. Wang et al.^26^ produced nanocomposite textiles consisting of boron nitride nanosheets and polyimide fibers with a thermal conductivity of ~ 13 Wm^−1^ K^−1^. Gong et al*.*^27^ reported an in-plane thermal conductivity of ~ 4 Wm^−1^ K^−1^ for graphene woven fabric-reinforced polymer composites. Our recent work^28^ showed that fabrics woven from ultra-high molecular weight polyethylene (UHMW-PE) fibers exhibit an in-plane thermal conductivity up to ~ 10 Wm^−1^ K^–1^. More recently, Tang et al.^29^ constructed electronic textiles using ultrasonication of non-woven fabric in a dispersion of carbon nanotubes, and measured a thermal conductivity of ~ 3 Wm^−1^ K^−1^. These prior studies demonstrate great promise of engineered textiles for heat spreading, which has also led to emerging of commercial fabrics in this material space. Considering this trend, there is a need for exploration of the interrelated thermal and mechanical properties of these commercially produced polymer-based materials for flexible heat spreading applications, and to obtain an understanding of their properties compared to conventional alternatives.

In this work, we explore high-performance polymer-based engineered textile materials with specific interest in their heat conduction behavior, while also considering relatively mechanically flexible materials. Based on a survey of commercially available materials, we first identified and acquired several commercial fabrics consisting of UHMW-PE (Dyneema) to characterize their properties that are relevant for wearable heat spreading applications. Specifically, the effective thermal conductivity and bending stiffness of these commercial Dyneema-based are measured using custom-developed thermal and mechanical metrology techniques. We also perform measurements for an in-house woven fabric constructed entirely from Dyneema fibers, as well as other conventional materials, in order to benchmark their performance. Further, we experimentally investigate the impact of creasing and thermal annealing of the fabrics on their effective thermal behavior to develop a better understanding of their potential for long-term use in wearable device applications.

## Materials survey and selection

In this section, the reported thermal and mechanical properties of commercially available polymer materials are surveyed, which serves as the basis for selection of the fabrics characterized in this study. Commercially available high-performance fiber materials that could potentially be integrated in advanced textiles are first identified. Then, a material having desirable properties for applications in wearable device thermal management is selected. Finally, existing textile/fabric products constructed from these fibers are acquired for subsequent characterization.

**Figure 1:**
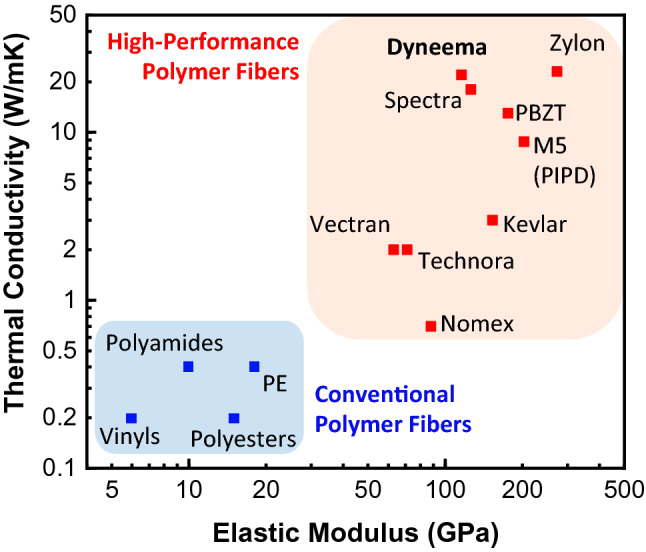
Elastic modulus and thermal conductivity of conventional polymer fibers (in blue) and commercially produced high-performance polymer fibers (in red). The high-performance polymer fibers typically have ~ 1–2 orders of magnitude higher thermal conductivity and elastic modulus (note the log scales). Data compiled from references^28,30–39^ with details for the high-performance fibers summarized in Table 1.

The two primary properties of interest are the elastic modulus and thermal conductivity. These properties are cataloged for various commercially produced high-performance fibers in Fig. 1, with details summarized in Table 1. Compared to conventional polymers, these materials have over an order of magnitude higher elastic moduli and thermal conductivities, typically greater than 50 GPa and 1 Wm^−1^ K^–1^, respectively. Among these materials, we identify Dyneema, a commercial gel-spun microfiber made of UHMW-PE, as a suitable starting material for the current study due to its standout thermal conductivity (20–30 Wm^−1^ K^−1^) and availability, in addition to good compatibility with large-scale textile manufacturing processes potentially enabling inexpensive production of advanced fabrics. The relatively lower elastic modulus of Dyneema as compared to other highest thermal conductivity materials, such as Zylon, is also an advantage in applications involving flexible substrates and devices.

**Table 1:** Details of the different high-performance polymer fibers (shown in Fig. 1).

Fiber	Fiber type	Fabrication method	Tensile modulus (GPa)	Refs.	Axial thermal conductivity (Wm^−1^ K^−1^)	Refs.	General properties
Dyneema	Ultra-high molecular weight polyethylene (UHMW-PE)	Gel-spinning process using spinneret (super drawing, heating, elongating, and cooling)			14.2	^30^	High strength to weight, abrasion resistance, mechanical strength, thermal conductivity
			101	^30^	23.6	^31^	
			113	^32^	28.4	^28^	
					22.5	^33^	
Spectra	Ultra-high molecular weight polyethylene (UHMW-PE)	Gel-spinning process	118	^30^	15.8	^30^	High strength to weight, abrasion resistance, mechanical strength, thermal conductivity
			113	^32^	20	^34^	
Zylon	Polybenzoxazole (PBO)	Spun from Polyphosphoric acid (PPA) solutions via dry-jet wet spinning	275	^30^	23	^30^	Very high mechanical strength, excellent heat resistance; very low thermal conductivity
			270	^35^	22.6	^31^	
PBZT	Poly (p-phenylene benzobisthiazole)	Dry-jet wet spinning	202	^30^	12.5	^30^	High modulus and high strength
			150	^36^			
PIPD (M5)	Polyhydroquinone-diimidazopyridine	Condensation polymerization followed by extrusion	76	^30^	8.8	^30^	High strength, modulus, and thermal stability
			330	^35^			
Kevlar	Aromatic polyamide (para aramid)	Liquid-crystalline polymer produced by wet/dry-jet wet spinning	120	^30^	3.1	^30^	High strength, toughness, thermal stability, flame resistance
			185	^35^			
Vectran	Aromatic polyester	Melt spinning/extrusion from liquid crystal polymer	60	^30^	1.7	^30^	High tensile and impact strength, abrasion resistance
			65	^35^	2.5	^37^	
Technora	Aromatic copolyamide (para aramid)	Condensation polymerization followed by spinning and drawing	71	^35^	1.98	^38^	High heat and chemical resistance, strength, and dimensional stability
Nomex	Meta aramid	Condensation reaction followed by spinning and drawing	88	^39^	0.65	^38^	Intrinsically flame resistant, high temperature resistant

**Figure 2:**
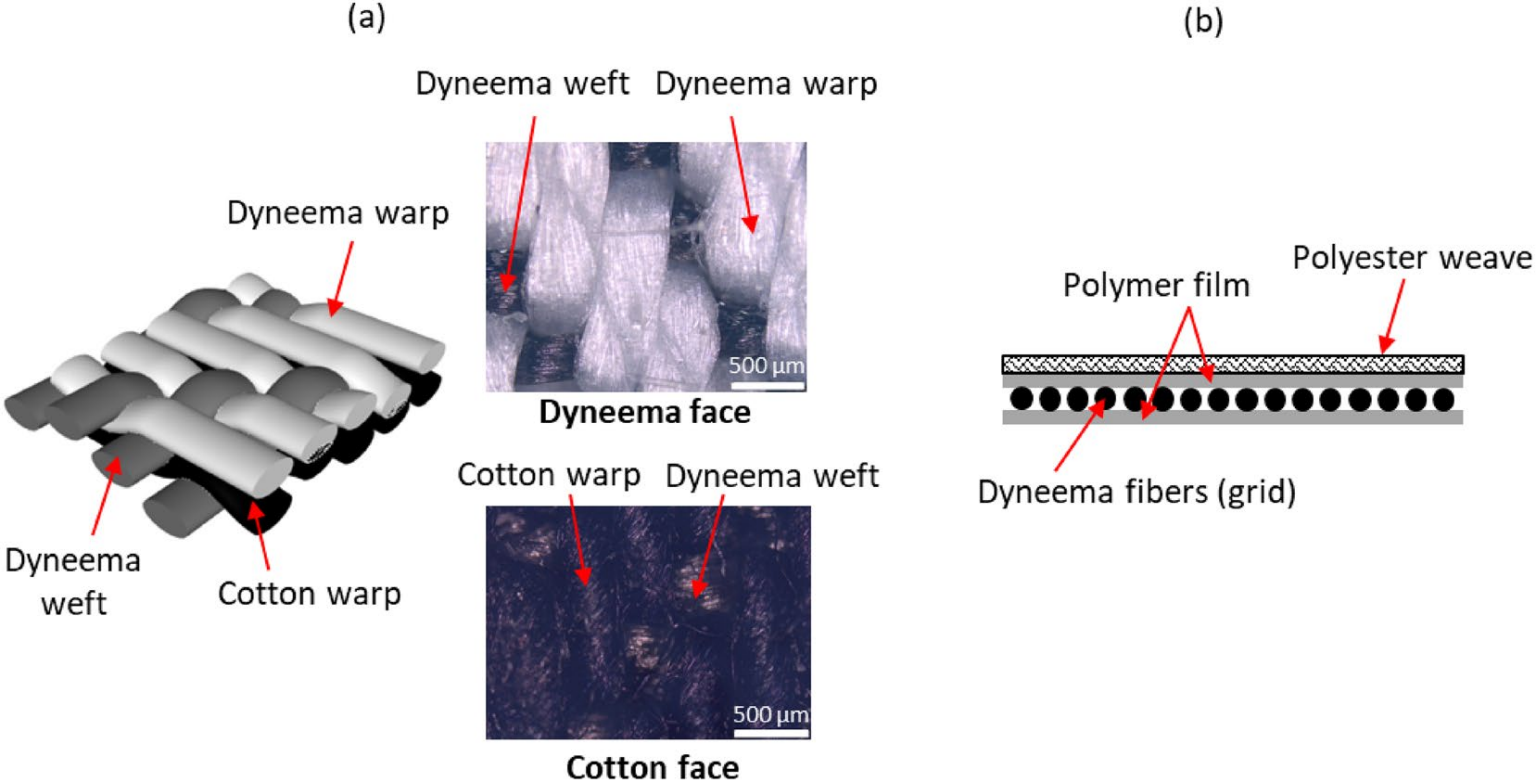
Representative schematics of the commercial Dyneema fabric samples: (**a**) the Dyneema denim fabric weave structure consisting of Dyneema and cotton yarns, with optical micrographs of the Dyneema and cotton faces for the Dyneema Black fabric shown as an example. (**b**) The Composite Dyneema fabric sample consisting of a grid of Dyneema fibers sandwiched between polymer films, with a polyester weave attached on one face.

The identified commercial Dyneema-based fabrics, the in-house fabricated sample constructed entirely from Dyneema fibers, and benchmarking materials considered in the present study are summarized in Table 2, along with a sample identifier used for each material. The commercial fabrics include two types of Dyneema denim fabrics (labelled “Dyneema Black” and “Dyneema BW”) and a Dyneema composite fabric (labelled “Dyneema Composite”) acquired from Rockywoods Fabrics LLC. As illustrated in Fig. 2a, the denim fabrics consist of a specially engineered double-warp weave configuration comprising Dyneema yarns along the warp and weft directions of the fabric, and cotton yarns along the warp. The composite fabric is made up of a grid of Dyneema fibers sandwiched between polymer films to form a UHMW-PE composite laminate, with a polyester weave is attached on one face (see Fig. 2b). The in-house fabricated sample (100% Dyneema) is a plain-weave Dyneema fabric constructed entirely from Dyneema SK75 microfibers (Atkins and Pearce Inc.) as described in our previous work^28^. Additionally, we acquire some conventional materials with no UHMW-PE content for benchmarking purposes: EeonTex, a non-woven fabric that is commonly used as a heater fabric in electronic textiles; a high-density polyethylene (HDPE) flexible sheet (McMaster-Carr); and a typical cotton twill weave fabric (tested for mechanical properties only).

**Table 2:** Summary of the materials considered in this study.

Material category	Sample identifier	Description
Dyneema (UHMW-PE) fabrics	100% Dyneema	Plain-weave fabric woven in-house, consists of Dyneema SK75 yarns along warp and weft directions, with 4–5 times higher yarn density along weft
	Dyneema black	Commercial double weave denim consisting of Dyneema and cotton warp yarns, and Dyneema weft yarns *(Supplier Specification: 62% Dyneema, 38% cotton)*
	Dyneema BW	Commercial double weave denim consisting of Dyneema and cotton warp yarns, and Dyneema weft yarns; some amount of polyamide and elasthane are present to provide stretch *(Supplier Specification: 52% Dyneema, 37% cotton, 9% polyamide, 2% elasthane)*
	Dyneema composite	Dyneema fiber-reinforced polymer composite with polyester weave *(Supplier Specification: 15% Dyneema, 65% polyester, 20% other polymer film)*
Benchmarking materials (no UHMW-PE)	EeonTex	Non-woven conductive e-textile heater fabric consisting of a polyester/nylon blend
	HDPE sheet	Bulk material sheet made of high-density polyethylene
	Cotton	Conventional cotton cloth

## Experimental methods

### Thermal metrology

**Figure 3:**
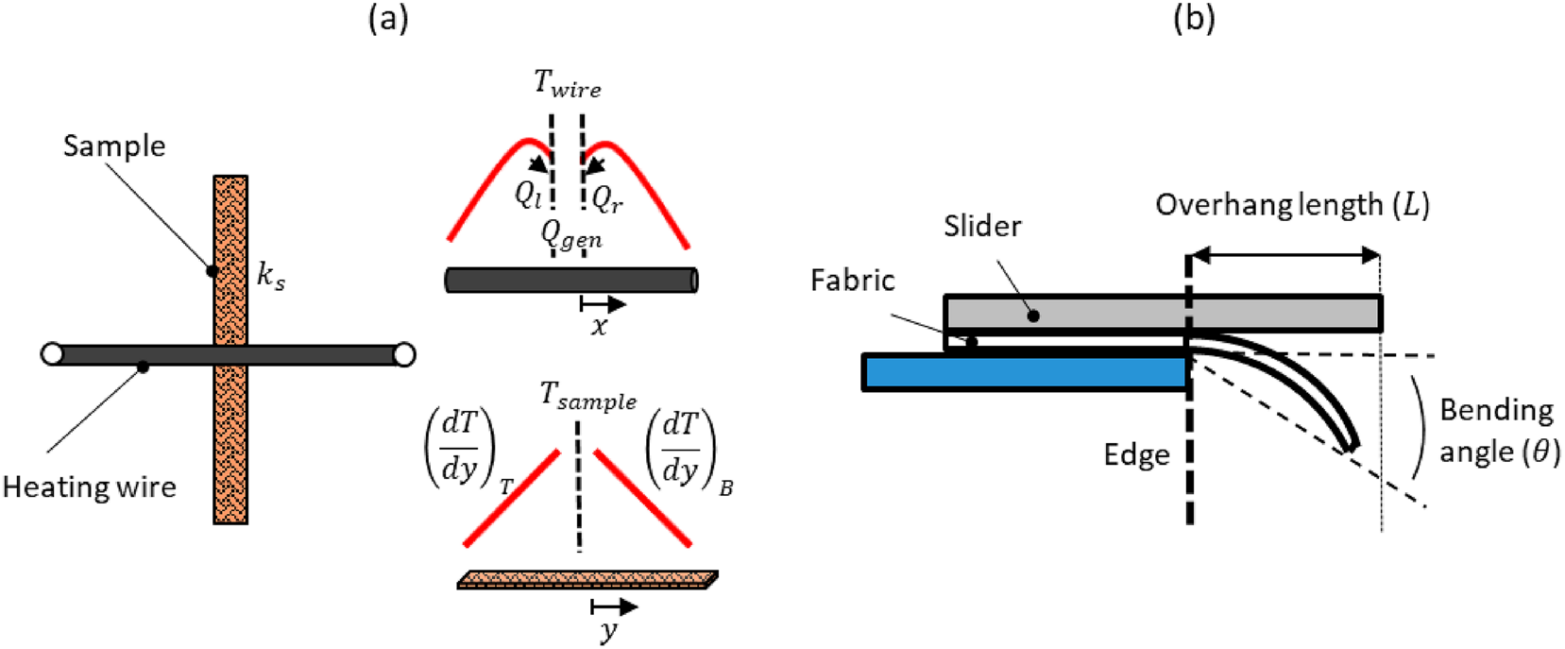
(**a**) Schematic representation of the thermal metrology setup showing a heating wire orthogonally oriented in contact with the test sample to be measured (left). Representative tempature profiles of the wire and sample shown (right) are captured using an infrared microscope. Heat rates ($${Q}_{l}$$ and $${Q}_{r})$$ are determined from the wire temperature profile, and the temperature gradients $$\left( {\left( {\frac{dT}{{dy}}} \right)_{T} \;{\text{and}}\;\left( {\frac{dT}{{dy}}} \right)_{B} } \right)$$ in the sample are used to calculate the sample thermal conductivity $${k}_{s}$$. (**b**) Illustration of the principle of the bend testing metrology, where the overhang length $$L$$ and bending angle $$\theta$$ of a fabric under its own weight enables quantification of the bending stiffness.

The in-plane thermal conductivity of the fabrics is measured using an in-house thermal measurement technique based on infrared microscopy. A brief description of this method is provided here and details of this technique are reported in our recent work^40^. As seen in Fig. 3a, the fabric sample is heated using a current-carrying nichrome wire placed orthogonally in contact with a strip of the fabric. The simultaneous measurement of the wire and fabric sample temperature profiles using a high-resolution infrared microscope (Quantum Focus Instruments) in a vacuum environment enables extraction of the effective in-plane thermal conductivity of the sample. This technique provides a robust approach to measure in-plane thermal conductivity based on the principle of simple energy balances, without the use of contact sensing methods, and eliminates the need to measure the thermal contact resistance between the heater wire and sample. For each fabric sample, five steady-state temperature datasets are recorded at different power levels (in the range of ~ 75–175 mW) to extract a single, average value of thermal conductivity so as to improve confidence and reduce uncertainty in the measured results. The approach for calculation of uncertainties in the measured thermal conductivity is described in our previous work^40^. Briefly, the uncertainties in the various experimental and modeling parameters are included following a standard uncertainty propagation analysis to calculate the total uncertainty in the heat flux and temperature gradient, and thereby the thermal conductivity of the sample. Ultimately, these measurements yield the in-plane thermal conductivity of the fabric samples.

### Mechanical metrology

To quantify the mechanical flexibility of the fabric samples, we characterize the bending stiffness (or flexural rigidity), which is a measure of the resistance to bending offered by the fabric material. We use an experimental approach for measuring fabric bending stiffness in accordance with the principle of the Pierce Cantilever Test and the ASTM D1388 standard^41^. For this purpose, a bending test fixture is designed and fabricated.

In this test, a fabric sample is placed under a weighted slider and manually slid over the edge of the fixture until the end of the fabric bends under its own weight to a prescribed angle (typically 41.5°). The bending stiffness of the fabric is quantified based on the overhang length and bending angle as illustrated in Fig. 3b. We note that the most recent version of the ASTM standard^41^ includes unexplained and ambiguous constants, as also discussed by Lammens et al*.*^42^. Therefore, in this work, the bending stiffness ($G$) (units of Nm) is calculated based on the traditional Pierce equation as follows:

$$G=W{C}^{3}$$

where $W$ is the weight per unit area (in N m^−2^) and $C$ is the bending length (in m). The overhang length ($L$) shown in Fig. 3b is measured to determine the horizontal length moved when the sample reaches the specified bend angle. This length is then used to calculate the bend length, *C*:

$$C=f\left(\theta \right)L,$$

where $\theta$ is the bend angle at which the overhang length is measured and $f(\theta$*)* is given by

$$f\left(\theta \right)={\left[\frac{\mathrm{cos}\left(\frac{\theta }{2}\right)}{8\mathrm{tan}\left(\theta \right)}\right]}^\frac{1}{3}.$$

The two key parameters, $L$ and $\theta$, are measured using scale rulers attached to components of the cantilever test fixture. To measure the overhang length ($L$), a weighted slider with an attached scale is always kept in contact with the fabric during the test. The displacement of the slider is the overhand length of the fabric, which is read from the scale at the end of the test. For the bending angle ($\theta )$, two sets of protractors are etched into transparent acrylic plates on both sides of the overhanging fabric sample. The uncertainties in this measurement are calculated via a standard propagation of errors in the measurement of the overhang length, $L$ (± 1 mm) and bending angle, $\theta$ (± 2°). Additionally, a validation of the measurement approach is provided in the “Supplementary Information S1” (Sect. S1.1) by comparing the measurements for a Kapton (polyimide) film to the calculated value from plate theory.

## Results and discussion

### Thermal conductivity

In this section, we report the results of the thermal conductivity characterization for the samples in Table 2. The HDPE, EeonTex, and Dyneema composite samples are isotropic in the in-plane direction, while the woven Dyneema denim and 100% Dyneema fabrics are anisotropic due to differing yarn materials and densities along the warp versus weft directions. For these fabrics, the thermal conductivity is measured along the high-density direction of constituent yarns, which is along the weft for the 100% Dyneema fabric and along the warp for the Dyneema denim fabrics.

**Figure 4:**
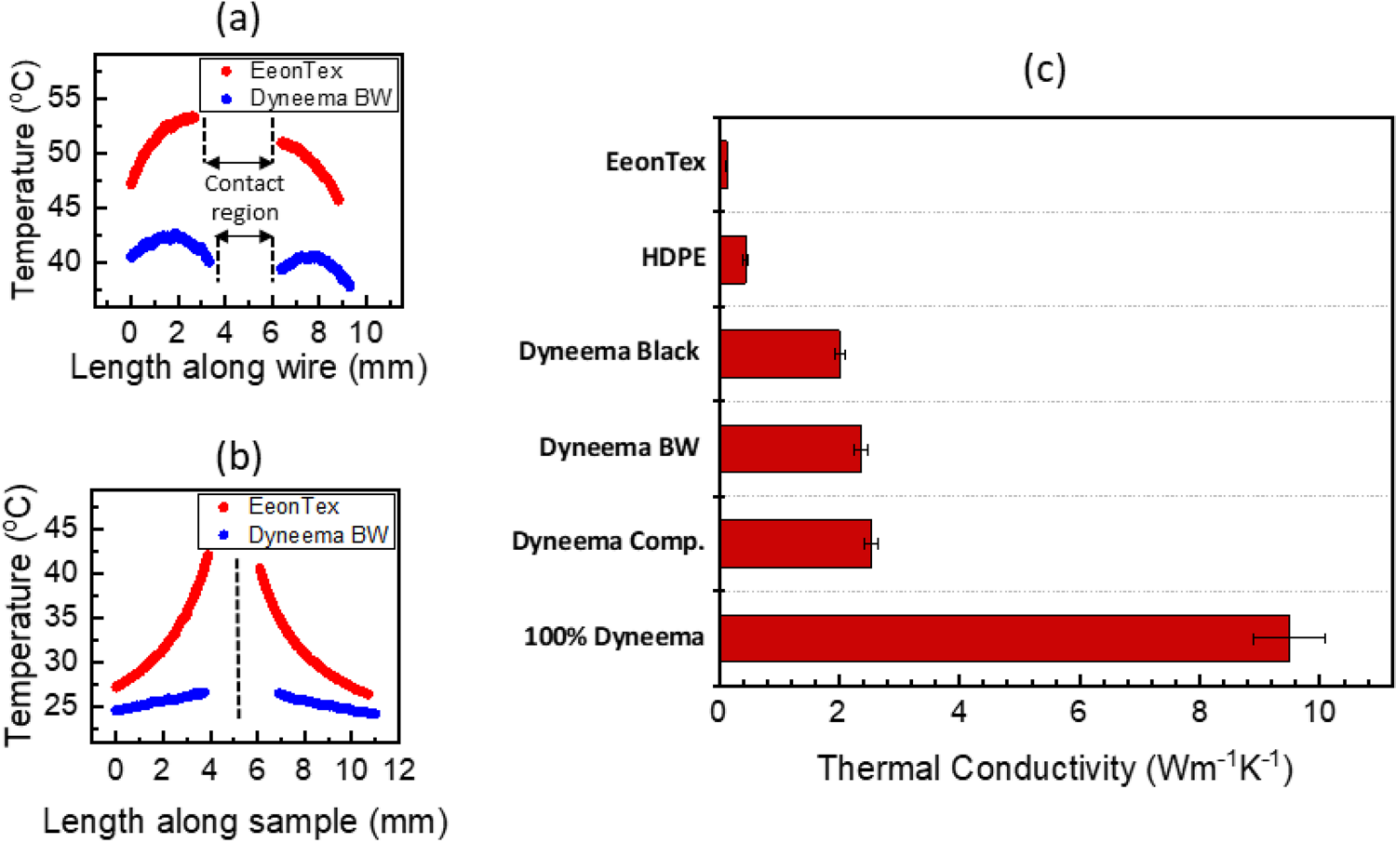
Representative temperature profiles of (**a**) the heating wire and (**b**) fabric sample obtained from IR thermal measurements of the Dyneema BW and EeonTex samples at the same electrical power input. The Dyneema BW sample exhibits significantly lower wire and sample temperatures, a steeper temperature gradient in the wire approaching the central region, and a relatively more linear sample temperature profile, which all are indicative of a higher in-plane thermal conductivity. (**c**) Measured effective in-plane thermal conductivity for the different samples.

During the thermal measurements, the in-plane heat spreading behavior of the sample can be qualitatively understood from the steady state temperature profiles. In the wire-sample arrangement, a higher thermal conductivity sample will dissipate more heat from the heater wire at the same input power. This is observed as a relatively steeper temperature drop in the wire in the direction toward the point of contact with the sample. Additionally, a higher thermal conductivity sample will exhibit a more linear temperature profile in the sample due to heat conduction along the sample dominating compared to radiation loss to the surroundings^28^. These heat transfer mechanisms (conduction and radiation) are of comparable magnitude for a low conductivity sample leading to a non-linear decaying temperature profile. These qualitative trends are illustrated in Fig. 4a,b, which show representative temperature profiles of the heater wire and the fabric sample for experiments with Dyneema BW and EeonTex samples at the same total input power of ~ 100 mW. The temperature of the heater wire (Fig. 4a) is significantly lower for the Dyneema BW fabric compared to the EeonTex, and the temperature gradient approaching the contact region with the sample is steeper. Further, the temperature of the sample (Fig. 4b) is also lower on average and has a near-linear profile for the Dyneema BW fabric, whereas there is a clear exponential trend in temperature (and higher average) for the EeonTex sample.

Based the measurements, the effective in-plane thermal conductivity for each sample is calculated, and these results are summarized in Fig. 4c. The commercial Dyneema-based fabrics have thermal conductivities in the range 2–2.5 Wm^−1^ K^−1^, and the 100% Dyneema fabric has a thermal conductivity of 9.5 Wm^−1^ K^−1^. This is a significantly higher conductivity compared to the HDPE sheet (0.45 Wm^−1^ K^−1^), and more than an order of magnitude higher than EeonTex (0.13 Wm^−1^ K^−1^), which is a typical electrically conductive textile material. Further, typical textiles such as cotton have a conductivity on the order of 0.05 Wm^−1^ K^−1^^43^, demonstrating remarkable potential for Dyneema-based fabrics to be integrated into wearable-device heat spreading applications.

### Bending stiffness

**Figure 5:**
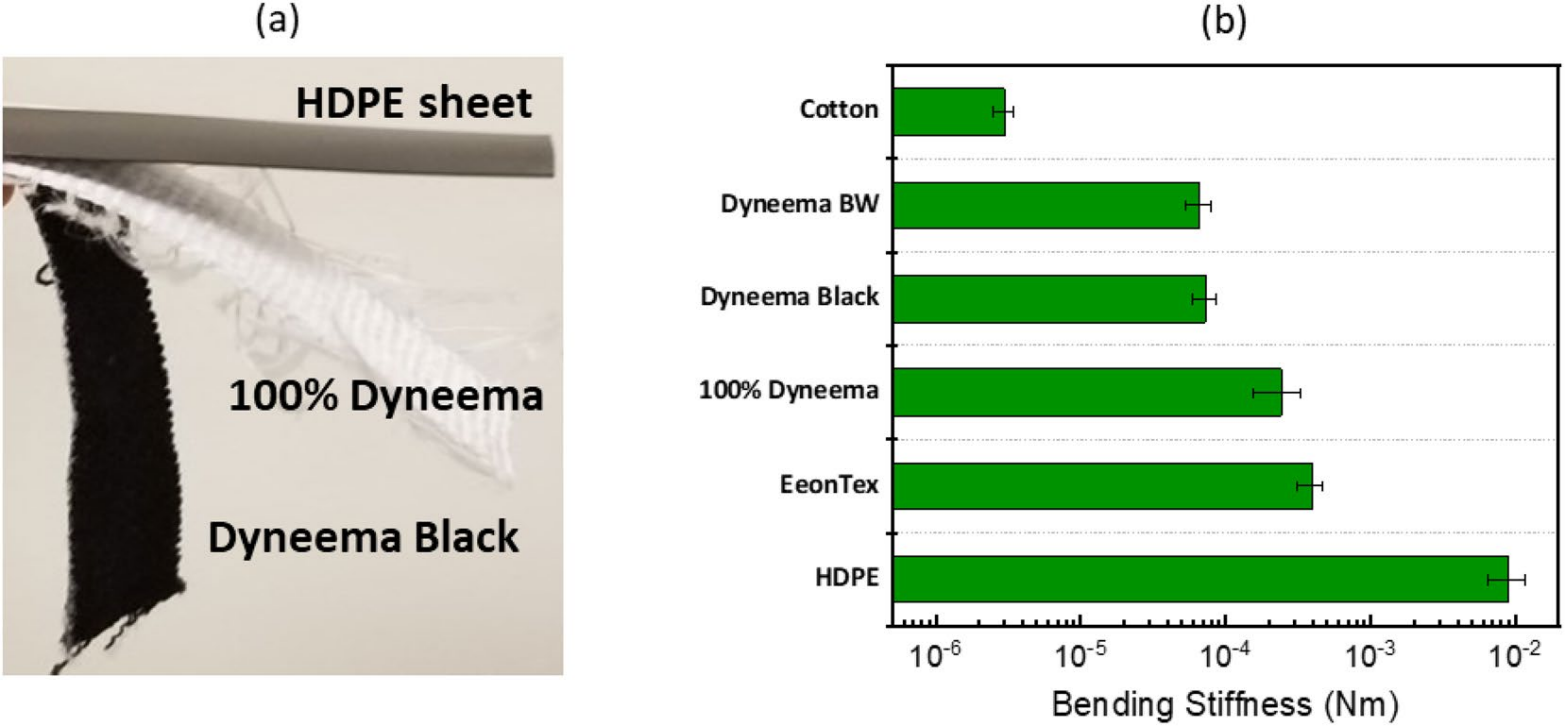
(**a**) Photograph of three different fabrics bending under their own weight. The stiff HDPE sheet does not visibly bend under its own weight, the Dyneema Black fabric is much more flexible as seen from its extent of bending, and the 100% Dyneema lies in between. (**b**) Measured average bending stiffness (flexural rigidity) based on the described bend testing metrology.

The bending stiffness provides a way of quantifying and comparing mechanical compliance and flexibility, which is an important consideration for wearable electronics-based substrates. Using the procedure described above, we quantify bending stiffness by measuring the overhang length at two different bending angles of 41.5° and 7.1°. The choice of these two angles is based on the value of $f\left(\theta \right)$, which is ~ 0.5 and 1 at these respective angles and typically in literature^41,42^. The results of the measurements are reasonably consistent at the two bending angles (See S1.2 in “Supplementary Information S1”). We note two extreme cases of measurement: the HDPE sheet being relatively stiff is tested only at a bend angle of 7.1° as the higher 41.5° bend angle is not achieved solely due to bending under its own weight even at high overhang lengths. In contrast, cotton being highly flexible reaches a high bending angle for a very small overhang length, and so it is measured only at the bend angle of 41.5°. Figure 5a shows a photograph that illustrates the relative flexibility of the HDPE film, 100% Dyneema fabric, and the Dyneema Black fabric.

The average value of the bending stiffness measured from the different bend angles is plotted in Fig. 5b, with higher values indicating lower flexibility. This measure spans over three orders of magnitude with the cotton sample and HDPE sheet representing the extremes. The Dyneema-based fabrics exhibit an intermediate level of flexibility and are more flexible than EeonTex.

### Crease testing

An important aspect related to flexibility is the durability of these fabrics (in terms of retaining their excellent thermal conductivity) as they will potentially undergo varying degrees of bending when used in practical applications. To evaluate durability under bending for the commercial Dyneema-based fabrics, the effect of creasing on the effective thermal properties is explored. Creasing, that is sharp folding of the fabric at a point by 180°, represents an extreme bending case.

**Figure 6:**
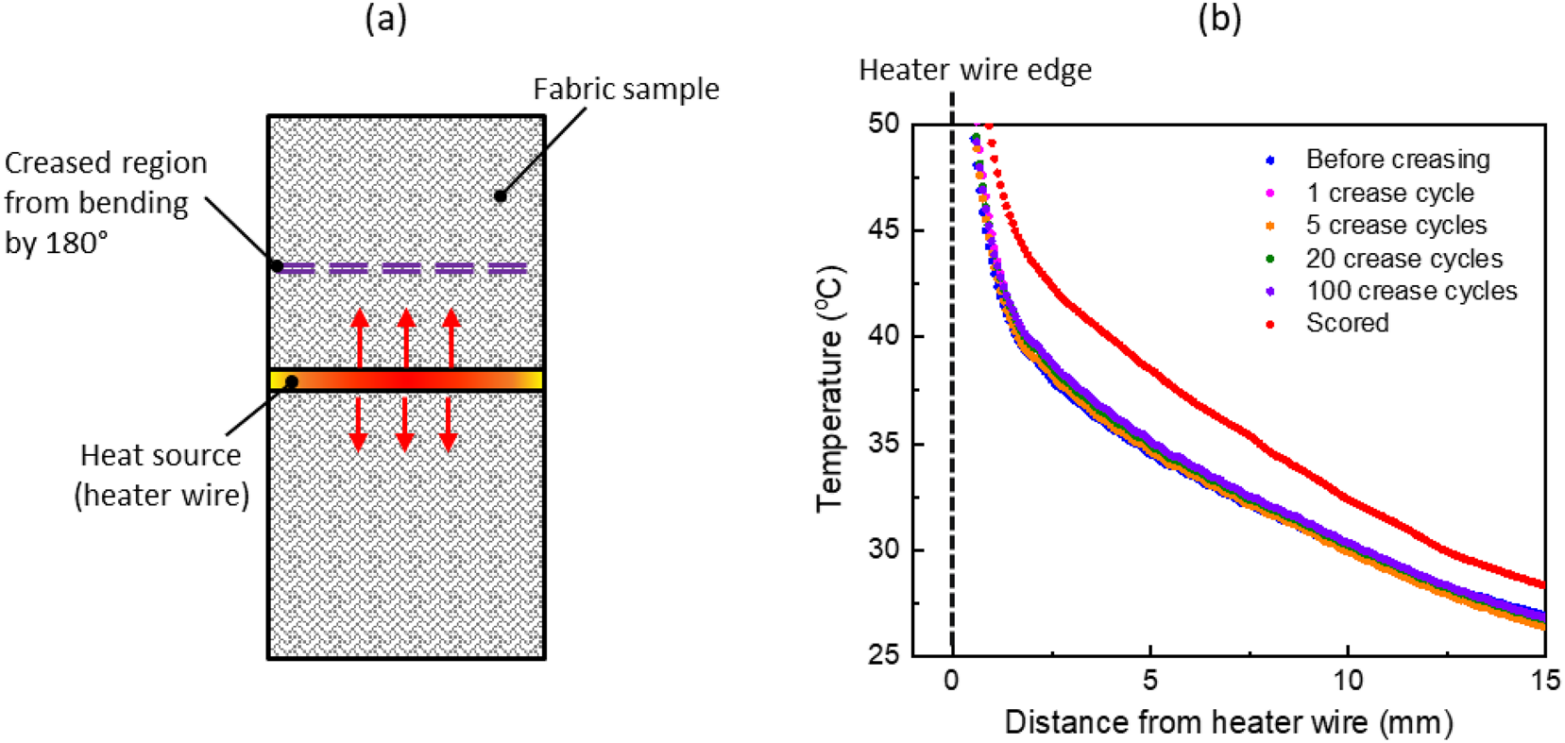
(**a**) Schematic diagram showing the top-view of the testing configuration to assess impact of creasing on thermal performance. The fabric sample is creased by bending by 180° at a specific location as indicated, and infrared temperature maps are captured before and after creasing. (**b**) Steady state temperature profile as a function of distance from the heater wire on the creased side of the fabric swatch, shown for increasing number of crease cycles and after scoring. The temperature profiles indicate no significant change in heat spreading properties except for the extreme scenario of scoring to purposely damage the individual yarns.

In these experiments, a swatch of fabric is suspended in a fixture and a nichrome heating wire is attached across the center of fabric. As illustrated in Fig. 6a, infrared thermal measurements are performed to view the temperature profile in the sample perpendicular to the wire before and after creasing the fabric at a specific location along its length. The creasing is achieved by folding the fabric by ~ 180° about this location while the fabric remains in place within the setup and then bending it back to the original position. The temperature is measured for the initial sample before creasing and after repeating the creasing multiple times (1, 5, 20, and 100 crease cycles). Steady state temperature maps shown in Fig. 6b, captured before and after multiple crease cycles indicate that the temperature profile along the fabric length is not significantly affected by creasing. As a point of reference, we perform a separate test to show that the temperature profile is significantly affected in an extreme case when the fabric is scored using a razor blade. We conclude from these observations that wrinkling or creasing the fabric, without damaging the weave structure or the individual yarns, will not significantly impact its effective thermal conductivity.

### Thermal annealing

Practical application of Dyneema-based fabrics in wearable devices must also consider the effect of exposure to high temperatures on the heat spreading performance of the fabrics. Previous studies on UHMW-PE fibers reveal a melting range of 147–152 °C^44,45^. We perform differential scanning calorimetry (DSC) measurements to verify that the melting ranges for as-received Dyneema SK75 fibers and the Dyneema Black denim fabric are in the expected range for UHMW-PE materials (see S2 in “Supplementary Information S1”). Also, other studies on UHMW-PE indicate respective long-duration and short-duration temperature limits of ~ 70 °C and ~ 100–130 °C^46^. These limits are based on evaluation of thermal stability at different aging times based on retention of mechanical properties such as modulus and tenacity. To the best of our knowledge, there has been no evaluation of the thermal stability of these materials as it pertains to the thermal conductivity.

To assess the impact of thermal annealing, we first anneal the Dyneema Black fabric samples in an oven (Binder ED 023) at different temperatures approaching the melting point (100, 115, 130, 145 °C) for 1 h, and perform DSC measurements after annealing. DSC can give a rough indication of the crystallinity of the sample by evaluation of the melting curves^47^, which directly relates to thermal conductivity, as the high thermal conductivity of UHMW-PE fibers is due to enhanced crystallinity and alignment as a result of the drawing process. Comparison of the DSC results after annealing with those of a control (as-received) fabric sample indicate that the DSC response does not change significantly for the annealing temperatures below 145 °C for a 1-h duration (see S2 in “Supplementary Information S1”). Therefore, we use fabric samples annealed at this highest 145 °C temperature condition for comparison of the in-plane thermal conductivity with a control fabric sample, using the measurement technique described previously.

The thermal conductivity of a single strip of control fabric is first measured in the in-plane test setup. Then, that same sample is annealed (145 °C for 1 h) and the thermal conductivity is measured again. The thermal conductivity of the fabric sample before and after annealing is measured to be 2.57 ± 0.1 Wm^−1^ K^−1^ and 2.12 ± 0.1 Wm^−1^ K^−1^, respectively. We note that, during this annealing process, the sample is very close to the melting point of Dyneema and undergoes some curling and deformation, even when the edges of the sample are constrained with tape. This is more pronounced for annealing of a thin strip (it is not observed for larger samples), and leads to fraying and unraveling of some of the yarns from the woven fabric, lowering the effective packing of yarns and thereby the effective thermal conductivity. Therefore, the measured 20% reduction in thermal conductivity can be regarded as an extreme scenario for the potential deleterious effects of annealing.

To eliminate these edge effects due to fraying and unraveling, a thin fabric strip cut out from a larger sample of annealed fabric (145 °C for 1 h) is also considered, and the thermal conductivity of this strip is demonstrated to be similar to that of a control (as received) fabric sample. This is facilitated using a simple experimental method that also enables qualitative visualization of the in-plane heat spreading of the two samples. In this approach, simultaneous transient and steady state IR temperature maps are captured for both samples (as received and annealed) when placed adjacent to each other and heated uniformly at one end. The relative in-plane thermal conductivity of the two samples is quantified based on fin heat conduction analysis (see S3 and “Video S1” in “Supplementary Information S1” for details). From these different thermal measurements, it can be concluded that exposure of the fabrics to high temperatures does not significantly impact their heat spreading ability for the conditions considered in this study.

### Thermal and mechanical property assessment

**Figure 7:**
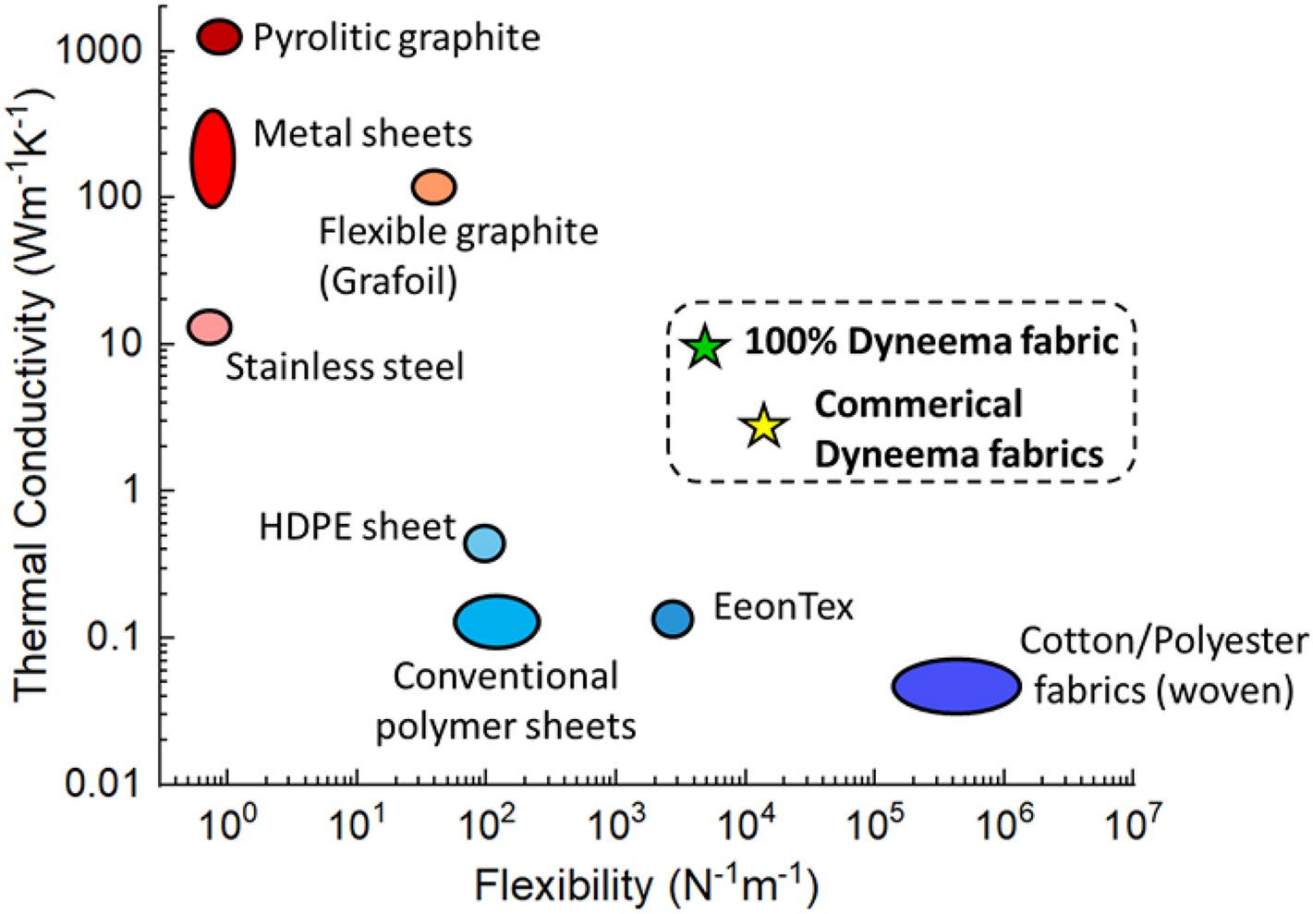
Thermal conductivity versus flexibility (inverse of bending stiffness) for different materials. The materials shown in shades of red (top left) are conventional heat spreaders and possess low flexibility, while those shown in shades of blue (bottom center-right) represent conventional polymer and fabric materials with high flexibility and low thermal conductivity. Dyneema fabrics (stars) break the trend by demonstrating significantly higher thermal conductivity relative to polymers, while retaining good mechanical flexibility. Data for standard materials (not characterized in this study) taken from references^48–52^.

It is valuable to assess the effective thermal and mechanical properties of the fabrics characterized in this study in the context of a broader set of materials that would be considered for similar applications. The inherent tradeoff between material thermal conductivity and flexibility is illustrated in Fig. 7, which catalogs these properties a broader set of standard materials that are typically used either for heat spreading or in wearable devices. The flexibility parameter shown here is the inverse of the bending stiffness. For the standard materials shown that are not characterized in this study, the bending stiffness is estimated using plate theory for a fixed thickness of 500 μm.

In general, conventional heat spreaders such as metals sheets and carbon-based materials possess low flexibility due to their high elastic modulus, while conventional polymer and fabric materials offer high flexibility at very low thermal conductivity. The UHMW-PE (Dyneema) fabrics stand as outliers that break free from this tradeoff in thermal versus mechanical properties. They possess significantly higher thermal conductivity relative to polymers and conventional textiles, while retaining similar levels of mechanical flexibility.

Another noteworthy finding is that the thermal conductivity of commercial UHMW-PE fabrics is similar in magnitude to that of novel high-performance fabrics in the literature, such as those produced by Gao et al.^25^, Wang et al.^26^, Gong et al.^27^, and Tang et al.^29^. These recent studies demonstrated that engineered woven or non-woven textiles fabricated using carbon-based materials, as well as high thermal conductivity nanocomposites, have thermal conductivities on the order of ~ 1–10 Wm^−1^ K^−1^. The present study shows that fabrics produced from oriented high-performance polymers have similar thermal conductivities with potential for further enhancement in the form of woven composites with high conductivity fillers. Further, the mechanical flexibility of such fabrics has been quantified, and the effect of mechanical creasing and exposure to high temperatures on the thermal properties have been characterized, which are important metrics that have not been investigated extensively for these or other materials in related literature.

## Conclusion

This work comprehensively characterizes the thermal and mechanical properties of commercial fabrics that are relevant for heat spreading applications in flexible and wearable device. Suitable high-performance polymer materials are surveyed, and a detailed review of relevant thermal and mechanical properties is provided. Dyneema fibers are identified as an ideal candidate for construction of heat spreading textiles and substrates, and the thermal conductivity and bending stiffness of fabrics constructed from these fibers are characterized using in-house thermal and mechanical metrology techniques, demonstrating great promise for such applications. Compared to other conventional heat spreading and textile materials of comparable thickness, UHMW-PE fabrics possess a unique combination of high thermal conductivity and mechanical flexibility suitable for these applications. Moreover, additional tests conducted with respect to both mechanical and thermal reliability assessment suggest that these materials can be used reliably in wearable device technologies. Overall, this study provides useful property data of high-performance polymers that can be leveraged for thermal and mechanical design studies related to high-performance wearable electronic packages. Additionally, our findings related to these previously unexplored properties of existing commercial materials pave the way for further research in the development of novel materials constructed from UHMW-PE to enhance key thermal and mechanical properties for application-specific performance criteria.

## Supplementary Information

Supplementary Information.

Supplementary Video 1.
