# Supplementary material for: Thermal and mechanical characterization of high performance polymer fabrics for applications in wearable devices
Source: Sci Rep. 2021 Apr 22;11:8705. doi: 10.1038/s41598-021-87957-7 (PMC8062592; doi:10.1038/s41598-021-87957-7)
Supplement: Supplementary file 1 — Supplementary Information. [file 41598_2021_87957_MOESM1_ESM.docx]

**Supplementary Information**

**Thermal and Mechanical Characterization of High-Performance Polymer Fabrics for Applications in Wearable Devices**

*Aaditya A. Candadai, Emily J. Nadler, Jack S. Burke, Justin A. Weibel, Amy M. Marconnet*

Birck Nanotechnology Center and School of Mechanical Engineering,

Purdue University, West Lafayette, IN 47907

**S1. Bending Stiffness**

**S1.1. Validation using Plate Theory**

An approximate validation of the bend testing measurement approach, similar to that adopted by Lammens *et al.* ^1^, is done by comparing the measured value for a Kapton (polyimide) film to the predicted bending stiffness using plate theory. For a solid film, the stiffness is given by:

$G=\frac{Et^{3}}{12(1-\nu^{2})}$ ,

where $E$ is Young’s Modulus (Pa), $t$ is the thickness of the material (m), and $\nu$ is Poisson’s ratio. Note that plate theory is not used as a theoretical comparison for the fabric samples measured in this study because it is applicable only for isotropic solid materials. The stiffness based on plate theory for the Kapton film is calculated to be $4.83\times{10}^{-4} Nm$, while the measured stiffness at a bending angle of 7.1° is $(2.7\pm0.8)\times{10}^{-4} Nm$. This comparison is also shown in Fig S1, alongside the other bending stiffness measurements. Considering that bending stiffness for the materials spans multiple orders of magnitude, this order of agreement between the theoretical value and the measured value is reasonable for the purposes of estimating and benchmarking the bending stiffness of the fabrics considered in this study.


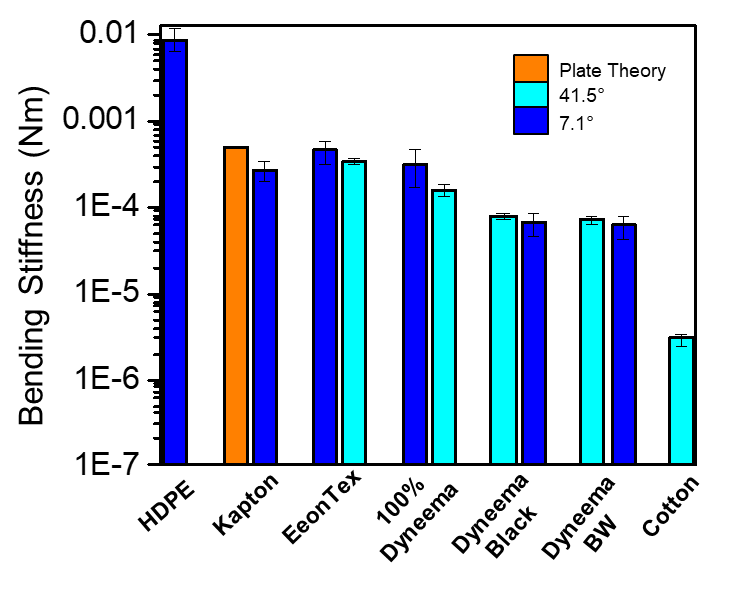


Figure S1: Measured bending stiffness (flexural rigidity) of all materials characterized at different bending angles. The measurements at and 7.1° and 41.5° are in reasonably good agreement.

**S1.2 Measurements at Two Bending Angles**

The measurement of bending stiffness is performed at two different angles for each sample (41.5° and 7.1°) except for HDPE and cotton as mentioned in the manuscript. The measured data are plotted in Fig S1 which demonstrates a reasonable agreement between the results at the two different bending angles.

**S2. Differential Scanning Calorimetry Measurements**

Differential Scanning Calorimetry (DSC) measurements are performed using a DSC system (NETZSCH DSC 2014 Polyma) to assess the impact of annealing of the Dyneema fibers and fabrics at different temperatures approaching the melting point. Fig S2a and S2b show the DSC response curves for Dyneema fibers and the Dyneema Black fabric, respectively, for as-received samples as well as for those subjected to annealing at different temperatures (100, 115, 130, 145°C) for 1 hr. From these plots, we see that the melting peak for the Dyneema fibers occurs at ~147°C, and that for the Dyneema denim fabric occurs at ~149°C. This is in good agreement with the expected melting range of 147-152°C for Dyneema ^2^.

**
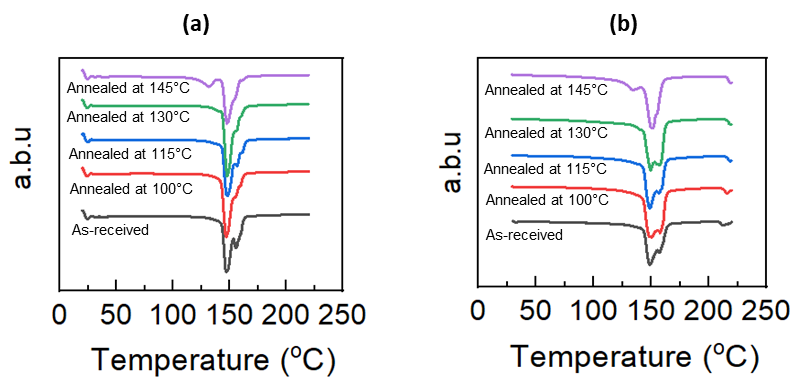
**

Figure S2: DSC response curves for (a) Dyneema fibers and (b) a Dyneema Black fabric, measured for as-received samples and for samples subjected to annealing at the different temperatures indicated. The early melting peak can be noted for the samples annealed at 145°C.

The results from Figure S2 also indicate no significant change in the DSC response for both fibers and fabrics, except for the samples subjected to annealing at the highest temperature condition of 145°C. For these samples (for both the fiber and the fabric), the onset of melting begins earlier as indicated by the short melting peak (~133-135°C) that precedes the more prominent, deep peak. This is because of the melting and recrystallization of a small fraction of polymer during and after the annealing process at 145°C. This fraction of polymer which is no longer highly crystalline and oriented then melts at a lower temperature during the DSC measurement, which is seen as an early, short melting peak in the response curve. Since the DSC response changes significantly only for the 145°C condition, the other temperature conditions are inferred to have no significant effect on the intrinsic crystallinity or thermal conductivity of the fabrics.

These DSC measurements guide our choice of using the fabric samples annealed at 145°C for 1 hour to assess the thermal stability of these materials as pertaining to the thermal conductivity, as reported in the manuscript.

**S3. Fin Heat Conduction Analysis**

A fin-based heat conduction analysis is used to obtain a relative measure of thermal conductivity of the Dyneema fabric before and after annealing. A Dyneema control fabric sample and an annealed fabric (at 145°C for 1 hour) are suspended between a heater block and cold block maintained at 20°C as shown in Fig S3. At a fixed heater power, steady state infrared (IR) temperature maps can be used to extract the relative thermal conductivity of the two samples by comparing the experimental temperature profile along the length of the sample and the theoretical temperature profile using the temperature distribution for a fin with prescribed tip temperature. For each sample, the theoretical temperature is given by ^3^:

$\frac{\theta(x)}{\theta_{b}}=\left( \frac{\left. {\left( \theta\right._{L}}/{\theta_{b}})\sinh\left( mx \right)+sinhm(L-x \right)}{\left. \sin h (mL \right)} \right)$ ,

where, for each sample (control or annealed), $\theta$ is the temperature rise (with respect to ambient) as a function of length $\theta_{b}$ is the difference between the base temperature (temperature at the heated end) and ambient temperature, $L$ is the sample length, $\theta_{L}$ is the difference between the prescribed tip temperature (obtained from the IR image) and the ambient temperature, and $m$ is a non-dimensional parameter given by $\sqrt{\frac{hP}{kA}}$ . Since the two fabric samples are cut to be of the same size and geometry, they have the same convection heat transfer coefficient $h$, perimeter $P,$ and cross-sectional area $A$. Using the above equation, the parameter $m$ can be individually obtained by fitting with the experimental steady state temperature profile as a function of length. Following this, the ratio of the thermal conductivities of the control and annealed samples ($k_{1}/k_{2})$ can be obtained. By shifting the location at which the base temperature is measured by a few pixels away from the heated end (up to ~1 mm away), four values of $k_{1}/k_{2}$ are calculated to provide a measure of uncertainty in this measurement. The calculated ratio ranges from ~0.81–1.06, indicating that the thermal conductivity of the control and annealed sample are estimated to be the same to within experimental uncertainty.


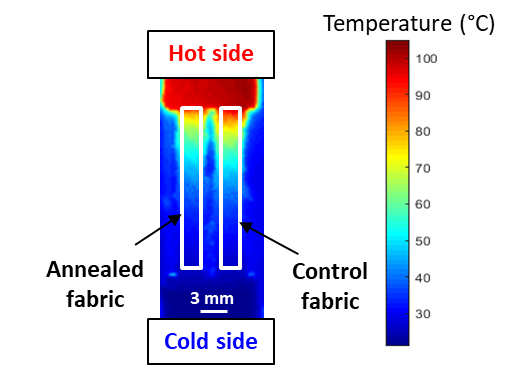


Figure S3: IR temperature image of the experimental test setup used to compare the relative in-plane thermal conductivities of a control fabric sample and an annealed (at 145°C for 1 hour) fabric sample.

In the attached video, a visual demonstration is also provided which illustrates the transient temperature distribution of both samples captured by the IR microscope after the heater is turned on. This provides a qualitative comparison of the in-plane heat spreading in both samples and indicates that there is no noticeable or significant change in the thermal conductivity.

**References**

1. Lammens, N., Kersemans, M. & Luyckx, G. Improved accuracy in the determination of flexural rigidity of textile fabrics by the Peirce cantilever test (ASTM D1388). *Text. Res. J.* **84** 1307-1314. (2014).

2. Teishev, A., Incardona, S., Migliaresi, C. & Marom, G. Polyethylene fibers-polyethylene matrix composites: Preparation and physical properties. *J. Appl. Polym. Sci.* **50**, 503–512 (1993).

3. Bergman, T., Lavine, A., Incropera, F. P. & Dewitt, D. *Fundamentals of Heat and Mass Transfer*. *John Wiley and Sons* **369**, (2013).
